# Changes in retail cigarette price after tax increase: Findings from the 2018–2020 ITC Vietnam surveys

**DOI:** 10.18332/tid/196675

**Published:** 2024-12-17

**Authors:** Thi Phuong Thao Tran, Thi Ngoc Phuong Nguyen, Thu Trang Vu, Thai Uyen Tran, Don Nguyen, Thi Hai Phan, Son Dao The, Min Kyung Lim, Anne C. K. Quah, Jin-Kyoung Oh, Hoang Van Minh

**Affiliations:** 1 Center for Population Health Sciences, Hanoi University of Public Health, Hanoi University of Public Health, Hanoi, Vietnam; 2 Department of Health Policy and Behavioral Sciences, School of Public Health, Georgia State University, Atlanta, United States; 3 Vietnam Tobacco Control Fund, Ministry of Health, Hanoi, Vietnam; 4 Center for Economics and Community Development, Hanoi, Vietnam; 5 Department of Social and Preventive Medicine, Inha University College of Medicine, Incheon, Republic of Korea; 6 Department of Psychology, University of Waterloo, Waterloo, Canada; 7 National Cancer Center, Graduate School of Cancer Science and Policy, Goyang-si, Republic of Korea

**Keywords:** cigarette price, cigarette tax, Vietnam, longitudinal study, low- and middle-income country

## Abstract

**INTRODUCTION:**

No longitudinal study has investigated the impact of cigarette tax increases on retail prices in Vietnam. This study aims to describe changes in the purchase price of cigarettes following an excise tax increase from 70% to 75% in January 2019.

**METHODS:**

Data were collected from people who currently smoke cigarettes in the longitudinal ITC Vietnam surveys: 1870 participants in Wave 1 (pre-increase), 1564 in Wave 2 (post-increase), and 1308 in Wave 3 (post-increase). Weighted mean self-reported prices of a cigarette pack (with standard error) were calculated for participants who were successfully followed up across three waves. These mean prices were calculated for domestic and international brands, categorized by specific cigarette brands. Percentage changes in mean prices were also measured, and significant differences in mean prices between follow-up waves (Waves 2 and 3) and the baseline (Wave 1) were assessed using paired t-tests. For brands with very small sample sizes, we used non-parametric tests, specifically the Wilcoxon signed-rank test instead of paired t-tests.

**RESULTS:**

The weighted mean price of a cigarette pack remained low and stable: VND 12330 (US$0.54) in 2018, VND 12700 (US$0.55) in 2019, and VND 12120 (US$0.53) in 2020 (1000 Vietnamese Dongs about US$0.04345, at 2018). International brands were substantially more expensive than domestic brands, but prices for both remained constant across all waves. Among domestic brands, Thang Long and Sai Gon showed slight price increases of around 3% and 5%, respectively (p<0.05). Among international brands, no statistically significant increase in mean prices was observed.

**CONCLUSIONS:**

The retail price of cigarettes remains low, indicating that the slight tax increase was insufficient to raise the current retail price significantly. Therefore, a substantial increase in cigarette prices by adding a specific tax is necessary.

## INTRODUCTION

Tobacco taxation is the single most effective policy intervention to reduce tobacco consumption, yet it has the lowest compliance among all tobacco control measures in Vietnam^1^. The country has applied a purely *ad valorem* excise tax system, calculated as a percentage of factory price^2^. Between 1993 and 2005, differential tax rates favored domestic over imported brands, facilitating cigarette smoking^2^. Since 2006, all cigarettes are subject to the same uniform *ad valorem* tax, which increased from 55% in 2006 to 65% in 2008. There was no change in tobacco tax rates for nearly a decade; from 2006 to 2016, it stood at 70%, and in 2019, it increased to 75%^3^. However, the current total tax (including VAT) remains low at only 34.3% of the retail price^4^, well below the WHO’s recommended 75%, and lower than most ASEAN countries and the global average level of 56%^5,6^. According to GATS 2015, the overall prevalence of tobacco use in Vietnam was 22.5%, with a much higher rate among men (45.3%) compared to women (1.1%)^7^.

To date, no longitudinal study has investigated the effect of cigarette tax increases on retail prices in Vietnam. Therefore, this study aimed to describe changes in the purchased price of cigarettes after a tax increase.

## METHODS

### Study design

The ITC Vietnam Survey is part of the International Tobacco Control Policy Evaluation Project (ITC) Project, which conducts longitudinal surveys in representative cohorts across 31 countries. In Vietnam, the survey was conducted in two districts of Hanoi: Cau Giay (representing urban areas) and Quoc Oai (representing rural areas), with an initial sample of 1988 people who smoke. This sample size was determined to be sufficient for detecting statistically significant changes while accounting for potential attrition, which also applied in other countries. The study followed standardized ITC Project protocols and quality control measures, with detailed methodologies, sampling techniques, and procedures described in technical reports^8-10^.

### Study population

Data were from the ITC in Vietnam: Wave 1 in 2018 (n=1988), Wave 2 in 2019 (n=2000), and Wave 3 in 2020 (n=1997)^8-10^. Wave 2 was conducted after an increase in cigarette excise tax from 70% to 75% in January 2019. Follow-up rates were 79.4% and 81.0% in 2019 and 2020, respectively. The study included people who currently smoked cigarettes across three waves: 1988 in Wave 1, 1701 in Wave 2, and 1545 in Wave 3, with 413 and 379 replenishments in Waves 2 and 3, respectively. After excluding participants with missing information on cigarette price or authenticity labels, and those buying single cigarettes, the final sample sizes were 1870 in Wave 1, 1564 in Wave 2, and 1308 in Wave 3.

### Measure

A face-to-face interview was conducted with participants and purchase price of a cigarette pack was asked using two questions: 1) ‘The last time you bought cigarettes, did you buy them by the carton, the pack, or as single cigarettes?’; and 2) ‘On average, how much did you pay for a carton of/pack of/single of cigarettes?’. All prices were adjusted for inflation using data from the General Statistics Office of Vietnam. The exchange rate used was 1000 VND to US$0.04345, according to The World Bank’s exchange rate in 2018. The brand of cigarettes at last purchase was examined. Cigarette brands were classified as domestic (e.g. Vinataba, Thanglong) or international (e.g. Marlboro, Craven).

### Statistical analysis

Weighted mean self-reported prices of a cigarette pack (with standard error) were calculated for participants who were successfully followed up across three waves. These mean prices were calculated for domestic and international brands, and categorized by specific cigarette brands. Percentage changes in mean prices were also measured, and significant differences in mean prices between follow-up waves (Waves 2 and 3) and the baseline (Wave 1) were assessed using paired t-tests. For brands with very small sample sizes, we used non-parametric tests, specifically the Wilcoxon signed-rank test instead of paired t-tests. In a sensitivity analysis, the mean self-reported prices of a cigarette pack were measured among all people who smoked across the three waves. Additionally, changes in self-reported cigarette prices were also measured among people who smoked and were successfully followed up between Wave 1 and Wave 2 (before and after the increase in cigarette tax) to increase the sample size.

Using cross-sectional sampling weights, we accounted for the uneven representation in particular age groups and regions, including attrition. Details are provided in reports^8-10^. All the analyses were performed by STATA Version 17.

### Ethics consideration

The study was approved by the Ethics Review Board for Biomedical Research, Hanoi University of Public Health, Vietnam (No. 419 & 422/2018/YTCC-HD3, No. 474/2019/YTCC-HD3, and No. 369/2020/YTCC-HD3) and the Office of Research Ethics, University of Waterloo, Canada (ORE#43707).

## RESULTS

Supplementary file Table 1 shows the characteristics of the study population across the three waves. Table 1 presents the weighted mean prices (in units of 1000 VND) of a cigarette pack among participants who smoked and were successfully followed up across three waves, categorized by brand. The overall mean pack prices showed minimal variation, with a 2.22% increase from 11.28 to 11.53 between Waves 1 and 2 (p=0.515), and a 1.33% increase from 11.28 to 11.43 between Waves 1 and 3 (p=0.698). Among domestic brands, Thang Long and Sai Gon showed slight price increases of around 3% and 5%, respectively (p<0.05). Sensitivity analysis comparing prices before and after the tax increase (Waves 1 and 2) showed no significant change overall, except for a 4% increase in Thang Long brand prices (p=0.001) (Supplementary file Table 2). Among international brands, there was no statistically significant increase in mean prices.

**Table 1:** Weighted mean price (in units of 1000 VND)^a^ of a cigarette pack among people who smoked and purchased a pack, in Wave 1, 2 and 3 of the ITC Vietnam surveys (N=651)^b^

*Brands*	*Total n*	*Wave 1*		*Wave 2*		*Wave 3*		*Percent change*			
		*Mean*	*SE*	*Mean*	*SE*	*Mean*	*SE*	*W2-W1*	p*	*W3-W1*	p*
**All brands in the sample^c^**	651	11.28	0.27	11.53	0.28	11.43	0.29	2.22	0.515	1.33	0.698
Domestic brands	580	10.01	0.17	10.23	0.15	10.18	0.16	2.20	0.331	1.70	0.482
Thang long	423	9.35	0.10	9.68	0.07	9.66	0.13	3.61	**0.006**	3.33	**0.062**
Vinataba	26	18.32	0.89	18.10	0.59	18.05	0.50	-1.20	0.838	-1.47	0.792
Sai Gon	20	9.84	0.10	10.36	0.20	11.47	0.38	5.26	**0.020**	16.54	**0.000**
Hoan Kiem	9	5.89	0.23	6.37	0.45	6.18	0.25	8.15	0.337	4.93	0.389
Du Lich	3	5.78	0.28	6.92	0.08	6.85	0.25	19.62			
Tam Dao	3	6.32	0.29	6.86	-	6.48	0.29	8.59		2.52	
Seal	5	9.73	0.22	9.81	0.00	9.56	0.16	0.81		-1.76	
Young star	2	10.00	-	9.41	0.15	9.70	0.00	-5.92		-2.97	
Thu Do	1	6.20	-	6.86	-	6.31	-	10.69		1.73	
Era	1	7.00	-	6.86	-	8.73	-	-1.96		24.76	
**International brands**	17	25.33	1.81	25.26	1.95	26.71	2.93	-0.28	0.978	18.48	0.683
555	4	29.83	2.40	30.99	1.42	38.16	3.76	3.89	27.92		
Malboro	6	22.15	1.32	20.80	0.95	20.59	0.94	-6.09		-7.04	
Horse	2	22.25	0.25	24.51	-	22.32	-	10.16		0.31	
Kent	2	25.00	-	26.47	-	26.47	0.39	5.88		5.88	
Craven	2	20.00	-	19.61	-	20.24	0.95	-1.95		1.20	
Captain black	1	65.00	-	63.73	-	63.07	-	-1.95		-2.97	

a VND: 1000 Vietnamese Dongs about US$0.04345, at 2018.

b Among all people who smoke that were successfully followed up.

c Among respondents whose last cigarette pack purchase was of the same brand at the three waves. Brand-specific estimates were assessed only among those who purchased the same brand in all three waves.

* Statistically significant differences in sub-groups for p<0.05.

Excluded were all people who smoke that had missing information on cigarette pack price in one of three waves. SE: standard error.

Supplementary file Figure 1 shows that cigarette pack prices remained relatively stable across the three waves. The weighted mean price per pack was VND 12330 (US$0.54), VND 12700 (US$0.55), and VND 12120 (US$0.53) in Waves 1, 2, and 3, respectively. International brands were substantially more expensive than domestic brands, but prices for both remained constant across all waves.

## DISCUSSION

The tobacco tax rate has remained very low in Vietnam. Our study shows that the 2019 excise tax increase from 70% to 75% of factory prices did not significantly impact retail prices. The weighted mean price of a cigarette pack remained low and stable: VND 12330 (US$0.54), VND 12700 (US$0.55), and VND 12120 (US$0.53) in 2018, 2019, and 2020, respectively. These prices are considerably lower than in other Western Pacific Region countries^11^. The pure *ad valorem* system has enabled tobacco companies to manipulate factory prices while maintaining retail prices, as evidenced by unchanged international brand prices despite the tax increase^12^. Additionally, evidence from other countries suggests that implementing a mixed system combining specific and *ad valorem* taxes, as successfully done in Thailand, could more effectively raise prices across all market segments while reducing price gaps between brands^13^. Currently, Vietnam’s cigarette prices are low because of low taxes. Tax policy has been proven to be the most effective strategy for reducing tobacco use demand, but it is the least implemented among MPOWER components and has shown the least improvement^14^. As of 2019, only 38 countries had increased taxes above 75% of the retail prices, while many low- and middle-income countries, including Vietnam, have extremely low tobacco tax rates or none at all^1^. Our study also supports previous evidence showing that cigarettes in Vietnam remain highly affordable^2,7^.

In 2020, the Ministry of Health, WHO, and partners proposed revising the Excise Tax Law, including two options to raise tobacco taxes: adding a specific tax of VND 2000–5000 per pack to the current 75% *ad valorem* rate, or increasing the *ad valorem* rate to 80% and later to 85%^2^. However, as of August 2024, neither option has been implemented, leading to the failure to achieve the National Strategy on Tobacco Control through 2020^2^. Consequently, the government has set a goal of reducing the smoking rate from 37% in 2025 to 32.5% in 2030 through the Vietnam Health Program for the period 2018–2030^15^, and proposed a new taxation roadmap in 2024 that extends to 2030. This plan involves adding a specific tax while keeping the *ad valorem* tax at 75%. The WHO and the Ministry of Health have suggested an aggressive strategy, beginning with VND 5000 (US$0.217) in 2026 to VND 15000 (US$0.652) by 2030. Vietnam has also identified tobacco industry interference in tax policy as a major obstacle to tobacco control^16^, contributing to the postponement of the proposed tax increases in 2020. Governments must prevent industry interference and adaptations, as well as crack down on illicit trade, to maximize the public health benefits of tobacco taxes. To maximize tobacco tax policy effectiveness, simplifying tax structures and relying more on specific excise taxes is crucial. Vietnam’s current pure *ad valorem* tax system poses challenges in implementation and weakens policy impact^17^. It complicates accurate price determination and allows for a wide price range among brands, enabling people who smoke to switch to cheaper options rather than quitting when taxes increase. Therefore, Vietnam should follow the lead of many other countries by moving away from a pure *ad valorem* tax system. Instead, it should adopt a more effective tax structure. This will strengthen the impact of the tobacco tax policy.

Our study revealed significant price variations between cigarette brands, with international brands costing nearly three times more than domestic brands. However, prices for both categories remained largely unchanged after the tax increase, except for a slight 4% increase in Thang Long brand. To maximize the impact of tobacco taxes on retail prices, a uniform specific tax system should be implemented.

### Limitations

While this study is the first longitudinal assessment of cigarette tax policy impacts in Vietnam using the ITC project’s standardized design, several limitations should be noted. The sample is not nationally representative, limiting generalizability. Self-reported cigarette prices may vary by purchase location. Price variations among sub-types within specific brands were not measured. Because of a limited number of participants using infrequent cigarette brands such as Du Lich, Tam Dao, and all other international brands, changes in cigarette prices for these brands could not be measured with sufficient statistical power.

## CONCLUSIONS

The retail price of cigarettes in Vietnam remains low, indicating that the slight tax increase was insufficient to raise the current retail price significantly. Therefore, a substantial increase in cigarette prices by adding a specific tax is necessary.

## Supplementary Material



## Data Availability

In each country participating in the Ιnternational Tobacco Control Policy Evaluation (ITC) Project, the data are jointly owned by the lead researcher(s) in that country and the ITC Project at the University of Waterloo. Data from the ITC Project are available to approved researchers, 2 years after the date of issuance of cleaned data sets by the ITC Data Management Centre. Researchers interested in using ITC data are required to apply for approval by submitting an International Tobacco Control Data Repository (ITCDR) request application and subsequently to sign an ITCDR Data Usage Agreement. The criteria for data usage approval and the contents of the Data Usage Agreement are described online (http://www.itcproject.org).
