# Supplementary material for: Changes in retail cigarette price after tax increase: Findings from the 2018–2020 ITC Vietnam surveys
Source: Tob Induc Dis. 2024 Dec 17;22:10.18332/tid/196675. doi: 10.18332/tid/196675 (PMC11650784; doi:10.18332/tid/196675)
Supplement: Supplementary file 1 [file TID-22-188-s1.pdf]

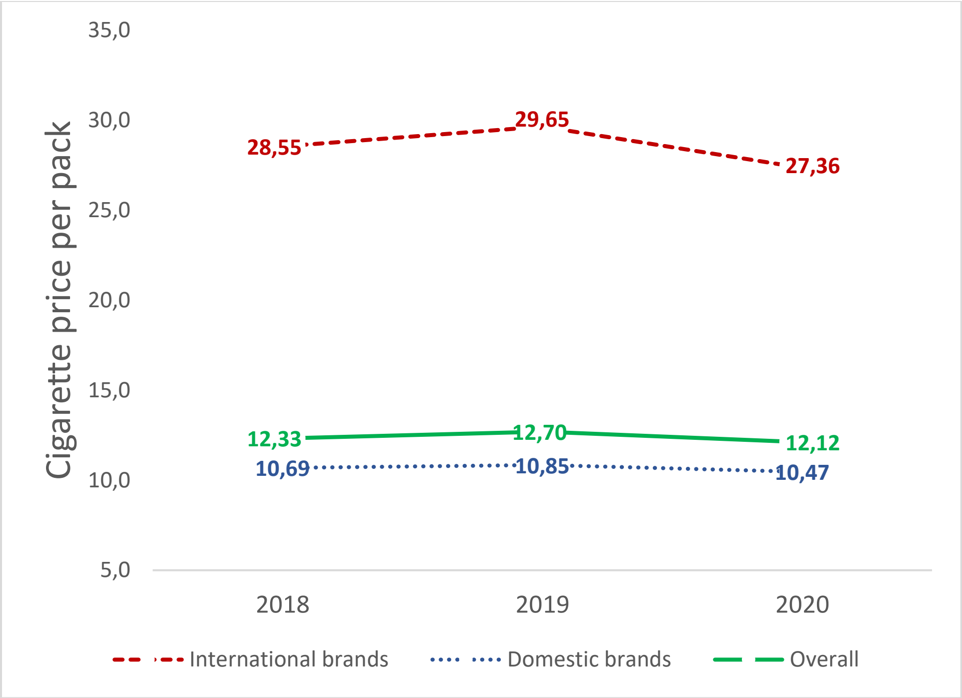

Supplementary Figure 1: Change in weighted mean price of a cigarette pack by brand over time. ***(Unit: VND1,000)***

Supplementary Table 1: Characteristics of study population by waves

	WAVE 1 (n=1870)		WAVE 2 (n=1564)		WAVE 3 (n=1308)	
N	n	weighted %	n	weighted %	n	weighted %
Age, mean (se)	46.10 (1.17)		46.06 (1.37)		46.34 (1.11)	
Age group						
18-24 years	115	6.81	81	6.43	80	6.33
25-34 years	368	19.14	306	20.20	241	21.40
35-44 years	395	20.44	342	20.81	293	22.02
45-54 years	423	20.70	353	21.73	269	19.98
55-64 years	400	23.28	344	21.78	287	20.11
>64 years	169	9.63	138	9.04	138	10.17
Region						
Urban	921	60.13	777	60.09	690	61.73
Rural	949	39.87	787	39.91	618	38.27
Highest education attainment						
≤ Primary school	217	10.15	197	11.12	155	11.64
Secondary school	597	31.48	486	30.84	415	31.05
High school	625	35.02	501	32.54	410	34.40
College/University +	431	23.35	380	25.50	328	22.90
Household monthly income						
Q1	470	20.84	401	21.65	352	26.10
Q2	539	28.87	401	26.68	290	21.31
Q3	395	21.97	504	32.42	401	30.93
Q4	443	28.32	256	19.26	254	21.66
Number of cigarettes smoked daily						
≤10	980	51.66	971	62.17	790	58.63
11-20	735	40.40	504	32.36	442	34.89
21+	155	7.94	256	5.47	76	6.47
Dual users of cigarette and other tobacco products						
No	1324	71.87	999	65.09	889	68.51
Yes	546	28.13	565	34.91	419	31.49
Cigarette brand of the last purchased cigarette*						
International brand	158	9.21	134	9.82	121	9.79
Domestic brand	1712	90.79	1420	90.18	1184	90.21
Form of last cigarette of purchase						
Carton	363	20.15	274	18.71	268	20.95
Pack	1507	79.85	1290	81.29	1040	79.05

**The domestic brands included Vinataba, Thanglong, Saigon, Tamdao, Hoankiem, Dulich, Thudo, etc., the international brands were Malboro, Craven, Camel, Kent, Jet, Raison, Esse, 555, etc.*

Supplementary Table 2: Weighted mean price of a cigarette pack among people who smoked and purchased a pack in Waves 1 and 2*

	n	Wave 1		Wave 2		% change	p-value
		Mean	SE	Mean	SE		
All brands in the sample**	1122	11.94	0.27	12.17	0.35	1.93	0.605
Domestic brands	1007	10.38	0.15	10.74	0.18	3.47	0.120
Thang long	756	9.40	0.08	9.78	0.08	4.10	**0.001**
Vinataba	64	18.40	0.60	18.62	0.57	1.20	0.794
Sai Gon	31	9.88	0.07	11.41	0.78	15.54	0.052
Hoan Kiem	11	5.81	0.22	6.32	0.41	8.81	0.268
Du Lich	7	6.32	0.37	7.45	0.28	17.87	
Seal	6	9.73	0.21	9.81	0.00	0.73	
Tam Dao	3	6.32	0.29	6.86	_	8.59	
Young star	3	10.00	0.00	9.61	0.19	-3.95	
Thu Do	2	5.52	0.59	5.75	0.96	4.20	
Era	2	7.00	_	8.55	0.47	22.13	
International brands	47	26.29	1.18	29.60	4.00	12.59	0.426
555	12	31.11	1.37	30.94	1.70	-0.55	0.939
Malboro	13	22.80	0.90	21.90	0.91	-3.95	0.486
Horse	4	23.09	0.89	23.52	0.89	1.86	
Kent	3	25.00	_	26.47	_	5.88	
Craven	2	20.00	_	19.61	_	-1.95	
Captain black	1	65.00	_	63.73	_	-1.95	

**among all people who smoke were successfully followed-up*
***among respondents whose last cigarette pack purchase was of the same brand at both waves. Brand-specific estimates were assessed only among those who purchased the same brand at both waves*
Excluding all people who smoke missing information on cigarette pack price in either Wave 1 or Wave 2
Unit of price: VND1,000

© 2024 Tran T.P.T. et al.
